# Biosynthesis of archaeal membrane ether lipids

**DOI:** 10.3389/fmicb.2014.00641

**Published:** 2014-11-26

**Authors:** Samta Jain, Antonella Caforio, Arnold J. M. Driessen

**Affiliations:** ^1^Department of Molecular Microbiology, Groningen Biomolecular Sciences and Biotechnology Institute, University of Groningen, GroningenNetherlands; ^2^The Zernike Institute for Advanced Materials, University of Groningen, GroningenNetherlands

**Keywords:** archaea, ether lipids, isoprenoids, biosynthesis, lipid divide

## Abstract

A vital function of the cell membrane in all living organism is to maintain the membrane permeability barrier and fluidity. The composition of the phospholipid bilayer is distinct in archaea when compared to bacteria and eukarya. In archaea, isoprenoid hydrocarbon side chains are linked via an ether bond to the *sn*-glycerol-1-phosphate backbone. In bacteria and eukarya on the other hand, fatty acid side chains are linked via an ester bond to the *sn*-glycerol-3-phosphate backbone. The polar head groups are globally shared in the three domains of life. The unique membrane lipids of archaea have been implicated not only in the survival and adaptation of the organisms to extreme environments but also to form the basis of the membrane composition of the last universal common ancestor (LUCA). In nature, a diverse range of archaeal lipids is found, the most common are the diether (or archaeol) and the tetraether (or caldarchaeol) lipids that form a monolayer. Variations in chain length, cyclization and other modifications lead to diversification of these lipids. The biosynthesis of these lipids is not yet well understood however progress in the last decade has led to a comprehensive understanding of the biosynthesis of archaeol. This review describes the current knowledge of the biosynthetic pathway of archaeal ether lipids; insights on the stability and robustness of archaeal lipid membranes; and evolutionary aspects of the lipid divide and the LUCA. It examines recent advances made in the field of pathway reconstruction in bacteria.

## INTRODUCTION

The “Woesian Revolution” in 1977 defined the three domains of life as the Eukarya, the Bacteria and the Archaea (Woese and Fox, 1977). The archaeal membrane lipid composition is one of the most remarkable feature distinguishing Archaea from Bacteria and Eukarya where the hydrocarbon chain consists of isoprenoid moieties which are ether linked to the enantiomeric glycerol backbone, glycerol-1-phosphate (G1P) in comparison to glycerol-3-phosphate (G3P) of bacteria and eukarya that is ester linked to the fatty acid derived hydrocarbon chain. Polar head groups on the other hand are common in all three domains of life. Other than this core archaeal diether lipid structure, a bipolar tetraether lipid structure is also prevalent in many archaea that span the entire archaeal membrane forming a monolayer (Koga and Morii, 2007). It should be stressed that ether-linked lipids are not unique to archaea *per se*, but are also found in Bacteria and Eukarya, although not ubiquitously distributed and usually only a minor component of the lipid membrane.

The stereo specificity of archaeal lipids and their unique structure was hypothesized to be chemically more stable thereby rendering the organism with the ability to resist and thrive in extreme environmental conditions (Koga, 2012). However, archaea are also found in mesophilic and neutrophilic environment where such a structural role of ether lipids is still not postulated. At the same time, the distinguishing lipid structures have formed the basis to the evolutionary studies describing archaeal and bacterial differentiation. Several models hypothesizing the early evolution of archaeal and bacterial phospholipid biosynthesis were proposed to answer intriguing questions about the nature of the ancestral membrane lipid composition (Lombard et al., 2012b). Understanding the archaeal lipid biosynthetic pathway is crucial to the above studies.

Decades of studies on the biosynthesis of archaeal lipids have advanced our knowledge on the major enzymatic processes but the pathway is, however still not completely understood. Several enzymes of the pathway have been studied and characterized biochemically but there are also gaps in our understanding of the archaeal lipid biosynthetic pathway and little is known about its regulation. With more genome sequences becoming available, advanced phylogenetic studies have been performed recently (Boucher et al., 2004; Daiyasu et al., 2005; Lombard et al., 2012a; Villanueva et al., 2014) and this helped to more precisely define its evolution. This review will focus on existing knowledge and recent studies on the enzymes of the pathway, the physicochemical properties of archaeal lipids, and the theories on the lipid divide.

## BIOSYNTHESIS OF ARCHAEAL MEMBRANE LIPIDS

### ISOPRENOID BUILDING BLOCKS AND CHAIN ELONGATION

**FIGURE 1:**
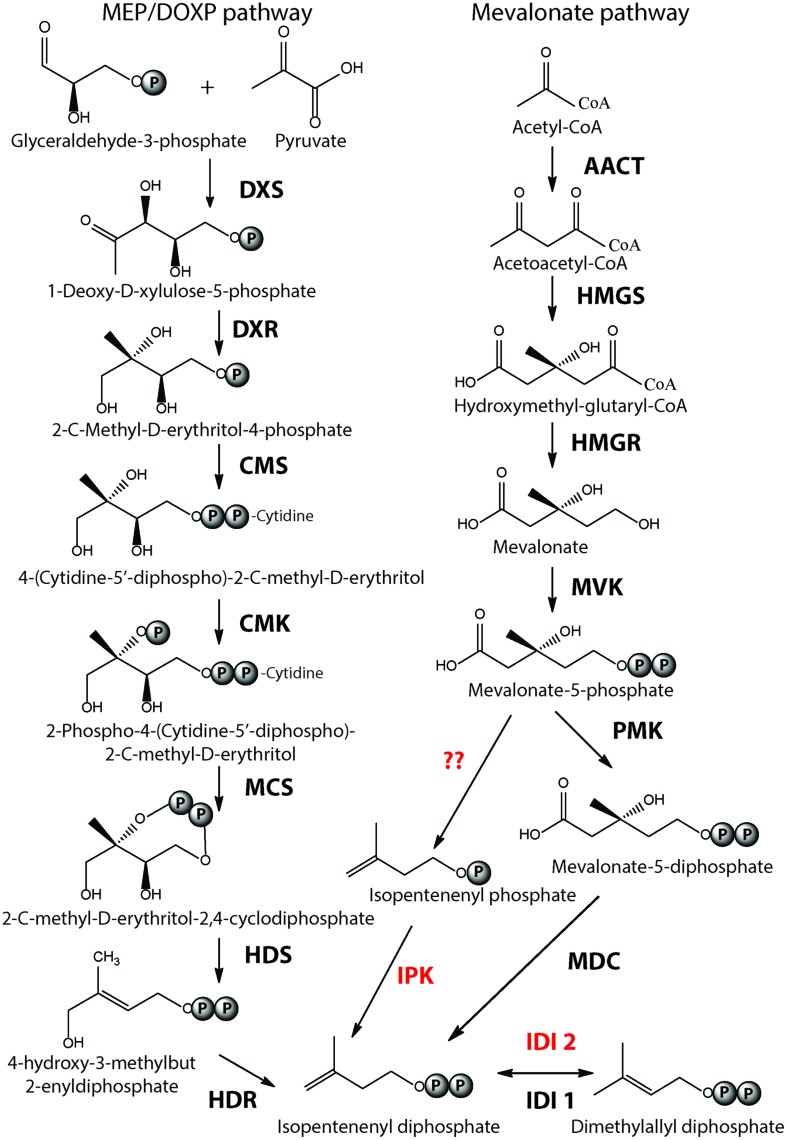
**Biosynthesis of Isoprenoid building blocks.** The three pathways leading to the synthesis of IPP and DMAPP – MEP/DOXP pathway, Mevalonate and the alternate mevalonate pathway (in red), which is prevalent in archaea are shown. Mevalonate and the alternate mevalonate pathways share four of their seven enzymatic steps. The enzyme acronyms are written in bold. DXS, 1-Deoxy-d-xylulose 5-phosphate synthase; DXR, 1-Deoxy-d-xylulose 5-phosphate reductase; CMS, 2-C-methyl-d-erythriol 4-phosphate cytidyltransferase; CMK, 4-diphosphocytidyl-2-C-methyl-d-erythritol kinase; MCS, 2-C-methyl-d-erythritol 2,4-cyclodiphosphate synthase; HDS, (E)-4-Hydroxy-3-methyl-but-2-enyl pyrophosphate synthase; HDR, (E)-4-Hydroxy-3-methyl-but-2-enyl pyrophosphate reductase; IDI, Isopentenyl diphosphate isomerase; AACT, acetoacetyl-CoA thiolase; HMGS, 3-hydroxy-3-methylglutaryl-CoA synthase; HMGR, 3-hydroxy-3-methylglutaryl-CoA reductase; MVK, mevalonate kinase; PMK, phosphomevalonate kinase; MDC, mevalonate-5-decarboxylase; IPK, isopentenyl phosphate kinase.

Isoprenoids are ubiquitous to all three domains of life. They are structurally diverse, forming more than 30,000 different compounds in nature ranging from steroids, quinones, carotenoids, and membrane lipids. The building blocks of isoprenoids are universal carbon five subunits called isopentenyl pyrophosphate (IPP) and dimethylallyl pyrophosphate (DMAPP) that are isomers. The biosynthetic pathway leading to the synthesis of IPP and DMAPP vary in different organisms (reviewed in Lombard and Moreira, 2011; Matsumi et al., 2011). To date, three pathways have been reported – 2-*C*-methyl-D-erythritol 4-phosphate/1-deoxy-D-xylulose 5-phosphate pathway (MEP/DOXP pathway) and two mevalonate (MVA) pathways. The MEP pathway genes share no homology to genes of the MVA pathway where pyruvate and glyceraldehyde-3-phosphate molecules are condensed together to form 1-deoxy-D-xylulose-5-phosphate (DXP) which is subsequently converted to IPP and DMAPP by five enzymes (Xue and Ahring, 2011; **Figure 1**). The MEP pathway is most common in bacteria although some Firmicutes possess the MVA pathway. The MVA pathway consists of seven enzymatic reactions where two acetyl-CoA molecules are condensed to form acetoacetyl-CoA, which is further condensed to form 3-hydroxy-3-methylglutaryl CoA (HMG-CoA). HMG-CoA undergoes phosphorylation and decarboxylation to form IPP via the formation of MVA (**Figure 1**). The classical MVA pathway is common to eukaryotes while some plants and photosynthetic eukaryotes possessing the MEP pathway in addition (Lombard and Moreira, 2011).

Interestingly, homologs of the last three enzymes of the classical MVA pathway, i.e., phosphomevalonate kinase, diphosphomevalonate decarboxylase and isopentenyl diphosphate isomerase could not be found in the majority of archaea (except in Sulfolobales that have classical MVA pathway; Boucher et al., 2004). This search led to the discovery of the alternate MVA pathway that differs from the classical one in the last three steps (**Figure 1**). The enzyme isopentenyl kinase (IPK) was first discovered in *Methanocaldococcus jannaschii* and found to be conserved in archaea (Grochowski et al., 2006). Its structure was determined (Dellas and Noel, 2010) and IPK enzymes from *Methanothermobacter thermautotrophicus* and *Thermoplasma acidophilum* were characterized biochemically (Chen and Poulter, 2010). In the alternate MVA pathway, phosphomelavonate is decarboxylated to isopentenyl phosphate by a decarboxylase (enzyme yet to be identified in archaea), which is subsequently phosphorylated to IPP by IPK. Furthermore, instead of the typical IDI1 isomerase that performs the last step of the classical MVA pathway, archaea have IDI2 which is not homologous to IDI1 but that performs the same reaction (Matsumi et al., 2011). Interestingly, a decarboxylase enzyme that converts phosphomelavonate to isopentenyl phosphate was found in green non-sulfur bacteria *Roseiflexus castenholzii* along with the presence of IPK enzymes indicating the existence of alternate MVA pathway in organisms other than archaea (Dellas et al., 2013). In general, IPP and DMAPP are synthesized by the MEP pathway in most of the bacteria and by two MVA pathways in eukarya and archaea. The classical MVA pathway of eukaryotes and the alternate MVA pathway of archaea share four of their seven steps.

The isoprenoid building blocks IPP and DMAPP undergo sequential condensation reactions where DMAPP acts as the first allylic acceptor of IPP leading to the formation of a carbon 10 (C10) compound termed geranyl diphosphate (GPP). Further condensation reactions proceed with the addition of IPP molecules where the chain length increases each time by a C5 unit forming farnesyl (C15), geranylgeranyl (C20), farnesylgeranyl (C25) diphosphate etc. This reaction of chain elongation is catalyzed by enzymes belonging to the family of prenyl transferases that are common to all three domains of life (Wang and Ohnuma, 1999; Vandermoten et al., 2009). Depending on the length and geometry of the final molecule, prenyl transferases can have several members in its family. The geometry of the molecule could be *cis* or *trans* and the chain length of the *trans* form generally ranges from C10 (e.g., monoterpenes) to C50 (e.g., Coenzyme Q10) and even longer for the *cis* forms. The chain length found in archaeal membrane lipids is always in the *trans* form and composed mostly of C20 [geranylgeranyl diphosphate (GGPP)] or C25 (farnesylgeranyl diphosphate). Tetraethers are composed of a C40 chain length, the synthesis of which is still unknown (discussed below). The archaeal prenyltransferase enzymes GGPP synthase and farnesylgeranyl diphosphate synthase synthesize specifically C20 or C25 product chain lengths, respectively (Ohnuma et al., 1994; Tachibana et al., 2000; Hemmi et al., 2002; Lai et al., 2009). They belong to short chain *trans* prenyl transferases family (that catalyze reactions ranging from C10–C25). Interestingly, a bifunctional prenyltransferase that catalyzes the synthesis of both C15 and C20 isoprenoids has been characterized from *Thermococcus kodakaraensis* (Fujiwara et al., 2004) and *Methanobacterium thermoautotrophicum* and is considered to be an ancient enzyme (Chen and Poulter, 1993). Multiple sequence alignment of homologues of the family display high sequence similarity with seven conserved regions where region two and seven contain the highly conserved aspartate rich sequences called first aspartate-rich motif (FARM) and second aspartate-rich motif (SARM) domains, respectively. Numerous mutagenesis and structural studies including several members of the family show that the region within the aspartate rich domains are involved in the binding and catalysis of the substrate while the regions flanking these domains are the major determinants of the chain length as they contribute to the size of the active site hydrophobic pocket. For example, GGPP synthase from *Sulfolobus acidocaldarius* could be mutated (at Phe-77 which is fifth amino acid upstream of FARM) to catalyze longer chain length (C30–C50) products (Ohnuma et al., 1996) and farnesyl pyrophosphate (FPP) synthase of *Escherichia coli* could be mutated (at Tyr-79, also 5th amino acid upstream of FARM) to create GGPP synthase (Lee et al., 2005). Physical factors have also been shown to influence the chain length of the product, e.g., the bifunctional enzyme farnesyl diphosphate/GGPP synthase of *Thermococcus kodakaraensis* shows an increase in the FPP/GGPP ratio with the reaction temperature (Fujiwara et al., 2004).

### GLYCEROL-1-PHOSPHATE BACKBONE

**FIGURE 2:**
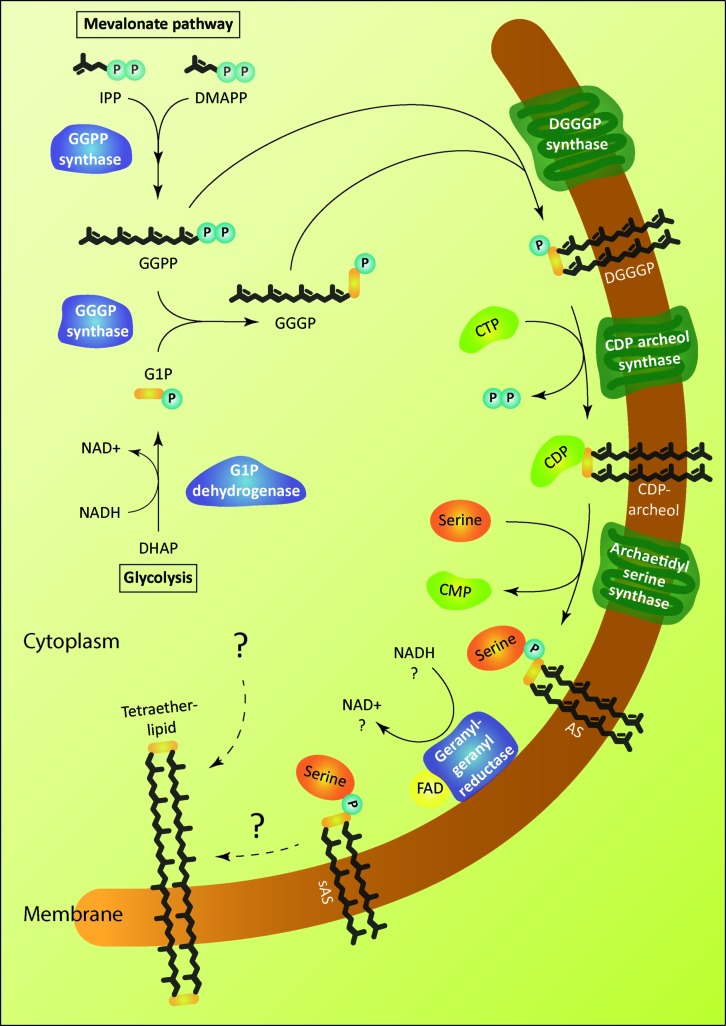
**Enzymatic pathway of archaeal lipid biosynthesis.** The soluble enzymes of the pathway are colored in blue and the membrane proteins in green. The biosynthetic steps leading to the formation of tetraether lipids is unknown. Archaetidylserine (AS) and saturated AS (sAS) are depicted as an example of polar head group modification. Isopentenyl pyrophosphate (IPP) and dimethylallyl pyrophosphate (DMAPP) are isomers. GGPP, geranylgeranyl diphosphate; G1P, *sn*-glycerol-1-phosphate; GGGP, 3-*O*-geranylgeranyl-*sn*-glyceryl-1-phosphate; DGGGP, 2,3-bis-*O*- geranylgeranyl-*sn*-glyceryl-1-phosphate.

The glycerophosphate backbone of archaea has an opposite stereoconfiguration than those of bacteria and eukarya. The archaeal enzyme responsible is G1P dehydrogenase that shares homology with alcohol and glycerol dehydrogenases but no homology to the bacterial/eukaryal G3P dehydrogenase. They belong to two separate families. However, both catalyze the reduction of dihydroxyacetone phosphate (DHAP) using nicotinamide adenine dinucleotide hydrogen (NADH) or nicotinamide adenine dinucleotide phosphate hydrogen (NADPH) as substrate (**Figure 2**; **Table 1**). G1P dehydrogenase uses Zn^2+^ for metal ion interaction in its active site (Han and Ishikawa, 2005) and transfers the pro-R hydrogen of NADH in contrast to G3P dehydrogenase that transfers the pro-S hydrogen (Koga et al., 2003); both enzymes bind the nicotinamide ring in an opposite orientation. G1P dehydrogenase is conserved in archaea. The enzyme has been purified and characterized from *Methanothermobacter thermoautotrophicus* as an octamer (Nishihara and Koga, 1997), *Aeropyrum pernix* as a homodimer (Han et al., 2002) and from *Sulfolobus tokodaii* (Koga et al., 2006). Its activity has been accessed in cell free homogenates of several archaea (Koga and Morii, 2007; Lai et al., 2009).

**Table 1:** Summary of kinetic parameters derived for enzymes of archaeal lipid biosynthetic pathway.

Name	Organism	Reaction	Kinetic parameters	Reference
*GGPP synthase*	*Methanobacterium thermoformicicum*	IPP + FPP → GGPP (60∘C)	K*_m_* IPP = 51.4 μM K*_m_* FPP = 28 μM V*_max_* = 437.9 nmol/min/mg	Tachibana et al. (1993)
*FPP/GGPP synthase*	*Thermococcus kodakaraensis*	IPP + DMAPP → GPP IPP + GPP → FPP IPP + FPP → GGPP (70∘C) IPP + DMAPP → GPP IPP + GPP → FPP IPP + FPP → GGPP (90∘C)	K*_m_* IPP = 23 μM, DMAPP = 9.5 μM K*_m_* IPP = 22 μM, GPP = 2.2 μM K*_m_* IPP = 3 μM, FPP = 1.7 μM K*_m_* IPP = 79 μM, DMAPP = 13 μM K*_m_* IPP = 31 μM, GPP = 3 μM K*_m_* IPP = 16 μM, FPP = 0.81 μM	Fujiwara et al. (2004)
*G1P dehydrogenase*	*Aeropyrum pernix*	DHAP+NADH → G1P (65∘C)	K*_m_* DHAP = 0.46 mM, *k_cat_* (min^-1^) = 154.25 K*_m_* NADH = 0.032 mM, *k_cat_* (min ^-1^) = 143.96	Han et al. (2002)
*GGGP synthase*	*Methanobacterium thermoautotrophicum*	G1P + GGPP → GGGP (55∘C)	K*_m_* G1P = 13.5 μM K*_m_* GGPP = 0.51 μM *k_cat_* (s^-1^) = 0.34	Soderberg et al. (2001)
	*Thermoplasma acidophilum*	G1P+GGPP → GGGP (55∘C)	K*_m_* G1P = 21.2 μM K*_m_* GGPP = 75 nM *k_cat_* (s^-1^) = 6.1, Vmax = 13.5 μmol/min/mg	Nemoto (2003)
*CDP-archaeol synthase*	*Archaeoglobus fulgidus*	DGGGP + CTP → CDP archaeol (65∘C)	K*_m_* DGGGP = 0.12 mM *k_cat_* (s^-1^) = 0.55	Jain et al. (2014)

Until recently it was thought that the stereo specificity is the hallmark of the ‘lipid divide’ where the G1P backbone is exclusively attributed to archaea. This was challenged by the discovery and characterization of the bacterial G1P dehydrogenase homolog of *Bacillus subtilis* which is annotated as ‘AraM’ (Guldan et al., 2008). It is also found in other related Gram positive and negative bacteria. Similar to the G1P dehydrogenase of *Aeropyrum pernix*, AraM forms a homodimer and performs G1P dehydrogenase activity. However, the two enzymes have different catalytic efficiencies and AraM is Ni^2+^ ion dependent (Han et al., 2002; Guldan et al., 2008). Remarkably, the G1P molecule eventually becomes part of an archaea type ether lipid heptaprenylglyceryl phosphate in *B. subtilis*, the function of which is still unknown (Guldan et al., 2011).

### ETHER LINKAGES

**FIGURE 3:**
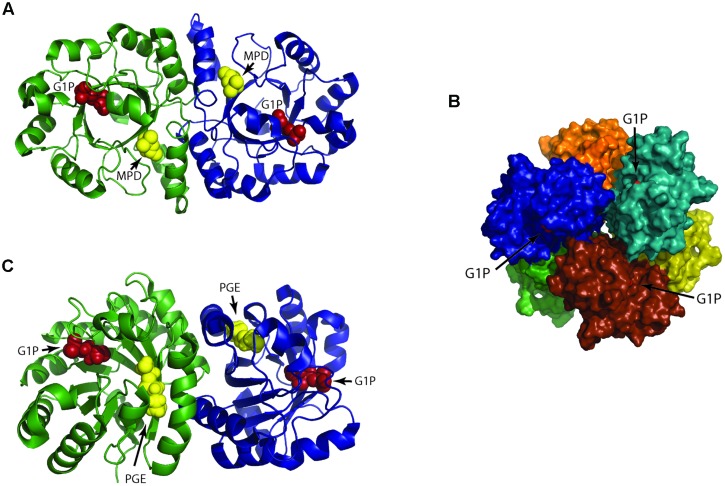
**Structure of 3-*O*-geranylgeranyl-*sn*-glyceryl-1-phosphate synthase (GGGP synthase). (A)** Crystal structure of group I dimeric GGGP synthase from *Archaeoglobus fulgidus* solved at 2.0 Å resolution (PDB: 2F6X) has a novel TIM-barrel modification (Payandeh et al., 2006). *Sn*-glycerol-1-phosphate (G1P) and a cryoprotectant (MPD) used in the experiment are colored as red and yellow spheres respectively. The cavity for MPD likely represents the binding site for the second substrate geranylgeranyl diphosphate (GGPP). **(B)** Crystal structure of group II GGGP synthase from *Methanothermobacter thermoautotrophicus* that forms a hexamer as a combination of three dimers was solved at 2.8 Å resolution (PDB: 4MM1; Peterhoff et al., 2014). G1P is displayed as red spheres. **(C)** A dimer from **(B)** is represented to compare with the dimeric group I GGGP synthase of **(A)**. The G1P and triethelene glycol (PGE) are colored as red and yellow spheres respectively. The hexameric subunits are rotated to each other unlike the dimers in **(A)**. The anchoring of G1P in group I and II takes place by the standard phosphate binding motif but their G1P binding pocket are different.

The first and second ether bonds between G1P and GGPP is catalyzed by the enzyme geranylgeranylglycerly diphosphate (GGGP) synthase and di-*0*- geranylgeranylglycerly diphosphate (DGGGP) synthase respectively. GGGP synthase is a conserved enzyme found in all archaea except Nanoarchaeota, which is a symbiont and possesses no genes of the lipid biosynthesis pathway (Podar et al., 2013). It is also found in some bacteria where the polyprenyl diphosphate substrate chain length could vary, e.g., PcrB of *Bacillus subtilis* which is a heptaprenyl diphosphate synthase (Ren et al., 2013). GGGP synthase is a crucial enzyme in the biosynthetic pathway of phospholipid metabolism in archaea as it brings together the three important characteristic features of the archaeal lipid structure – stereoisomeric G1P glycerol backbone and isoprenoid GGPP side chain linking them together via an ether bond (**Figure 2**; **Table 1**). Phylogenetic analysis of the GGGP synthase enzymes distinguishes it into two families, group I and group II, both comprising of archaeal and bacterial sequences. Several enzymes from both the groups have been characterized and a recent study performed the biochemical analysis of 17 members of GGGP synthase family (Peterhoff et al., 2014). The enzymes of group I form dimers (except the monomeric GGGP synthase of *Halobacterium salinarum*) and the group II enzymes are dimeric or hexameric in nature. Both the groups are further subdivided into Ia, Ib, IIa, and IIb with a and b corresponding archaea and bacteria, respectively. Crystal structures of enzymes from all the four groups have been solved. The first crystal structure from the group I GGGP synthase of *Archaeoglobus fulgidus* displays a modified triose phosphate isomerase (TIM)-barrel structure (Payandeh et al., 2006). It forms a dimer bound to the G1P substrate with a central eight-stranded parallel β-barrel and a hydrophobic core surrounded by α-helices (**Figure 3A**). Helix-3 is replaced by a ‘strand’ which is a novel TIM-barrel modification not observed previously. The substrate GGPP binds to the deep cleft traversing the top of the β-barrel. There is a ‘plug’ at the bottom of the barrel and the active site lies at the C-terminal end. The G1P molecule sits near the top inner rim of the barrel and the phosphate group binds to the standard phosphate-binding motif of the TIM-barrel. G1P forms 14 hydrogen bonds within the active site. The (βα)8-barrel fold is found in all the other structures of the GGGP synthases as well with the active site at the C-terminus. The crystal structure of group II archaeal hexameric GGGP synthase of *Methanothermobacter thermoautrophicus* displays a combination of three dimers that resemble the group I dimer (**Figures 3B,C**). In group II, however, the plug of the barrel is longer than in group I and there are ‘limiter residues’ that restrict the length of hydrophobic pocket to accommodate the polyprenyl diphosphates of a specific length. Interestingly, an aromatic anchor residue is responsible for the hexameric configuration of the enzyme, mutation of which causes it to dimerize without any loss of activity (Peterhoff et al., 2014).

The intrinsic membrane protein DGGGP synthase catalyzes the formation of the second ether bond between the substrate GGGP and GGPP to form DGGGP (**Figure 2**). It belongs to the family of ubiquinone-biosynthetic (UbiA) prenyltransferases, the members of which are responsible for the biosynthesis of respiratory quinones, chlorophyll, heme etc. by transferring a prenyl group to the acceptors that generally have hydrophobic ring structures. DGGGP synthase is divergent among archaea and could not be identified in the genomes of Thaumarchaeota (Villanueva et al., 2014).

Unlike other enzymes of the pathway, DGGGP synthase has not been well characterized probably due to technical limitations with overexpression of the membrane protein. The DGGGP synthase activity was first found in the membrane fraction of *Methanothermobacter marburgensis* (Zhang and Poulter, 1993). Later the gene was identified in the genome of *Sulfolobus solfataricus* as UbiA-2, cloned in *E. coli* and purified to study the Mg^2+^ dependent enzymatic activity using radiolabeled substrates and mass spectrometry (Hemmi et al., 2004). The ratio of the substrates utilized in the reaction was found to be 1:1.1 in a double labeling experiment using [^3^H]GGPP and [^14^C]GGGP, respectively. Specificity for GGPP and GGGP was also measured by substituting them with different prenyl substrates, of which none of them were used in the reaction by DGGGP synthase. In another study, DGGGP synthase was shown to accept both the S and R form of GGGP showing that unlike GGGP synthase, it is enantio unselective (Zhang et al., 2006). DGGGP synthase activity of *Archaeoglobus fulgidus* (Lai et al., 2009) and *Methanosarcina acetivorans* (Yokoi et al., 2012) was also observed in *E. coli* when the corresponding genes were expressed along with four previous enzymes of the pathway. However, the expression level of the enzyme was either too low to detect (Lai et al., 2009) or not investigated (Yokoi et al., 2012). In a later study, a higher expression level of DGGGP synthase of *Archaeoglobus fulgidus* was obtained in *E. coli* by changing the ribosome-binding site and the activity of purified DGGGP synthase was monitored (Jain et al., 2014).

### CDP ARCHAEOL FORMATION

The next step in the archaeal lipid biosynthetic pathway is the activation of DGGGP by cytidine triphosphate (CTP) to form the substrate for polar head group attachment called cytidine diphosphate (CDP)-archaeol (**Figure 2**; **Table 1**). The reaction is brought about by the enzyme CDP-archaeol synthase (CarS), the activity of which was first studied in the membrane fraction of *Methanothermobacter thermoautotrophicus* (Morii et al., 2000). Using various synthetic substrate analog, the activity was found to be specific for unsaturated archaetidic acid with geranylgeranyl chains and did not depend on the stereo specificity or ether/ester bond of the substrate. Minute amount of CDP-archaeol were also detected in growing cells labeled with inorganic ^32^P. The gene responsible for this activity was only identified in a recent study (Jain et al., 2014). The enzyme CarS is conserved among archaea (except Nanoarchaeota). However, like the enzyme DGGGP synthase, it could not be identified in the families of Thaumarchaeota.

Interestingly, an analogous reaction is found in the bacterial phospholipid biosynthetic pathway where phosphatidic acid is activated by CTP to form CDP diacylglycerol by the enzyme CDP diacylglycerol synthase (CdsA). Although the sequence similarity between CdsA and CarS is very low, hydropathy profile alignment of the two families shows similarity in their secondary structure with overlapping transmembrane segments and cytoplasmic loop regions residing in the C-terminus half. CarS from *Archaeoglobus fulgidus* was expressed and purified from *E. coli*. Similar to CdsA, CarS activity was found to be dependent on Mg^2+^, both accepts CTP and deoxycytidine triphosphate (dCTP) as substrates and does not utilize adenosine triphosphate (ATP), guanosine triphosphate (GTP), or thymidine triphosphate (TTP) nucleotides in the reaction using substrate DGGGP. However, the two enzymes displayed distinct activity with respect to the lipid substrate specificity where CarS only accepts unsaturated archaetidic acid with geranylgeranyl chains, while CdsA takes phosphatidic acid (Jain et al., 2014).

### POLAR HEAD GROUP ATTACHMENT

The polar head groups serine, ethanolamine, glycerol and *myo*-inositol are found in the phospholipids in all three domains of life. The enzymes that catalyze the replacement of the cytidine monophosphate (CMP) entity of CDP-archaeol or CDP-diacylglycerol with a polar head group are homologous and belong to CDP-alcohol phosphatidyltransferase family (Koga, 2011). Archaetidylserine (AS) synthase catalyzes the formation of AS from CDP-archaeol and L-serine (**Figure 2**) and is homologous to bacterial phosphatidylserine (PS) synthase. The enzyme can be classified into two subclasses. Subclass I includes enzymes distributed in Gram-negative bacteria, such as *E. coli* while subclass II enzymes are widespread among Gram-positive bacteria (*B. subtilis*), yeast and archaea. Studies using cell free extracts of *Methanothermobacter thermautotrophicus*, *B. subtilis*, and *E. coli* showed that both the AS and PS synthase from *Methanothermobacter thermautotrophicus* and *B. subtilis* have a broad substrate specificity and can accept lipid derivatives from archaea or bacteria. On the other hand, the *E. coli* PS synthase was specific for bacterial lipid derivatives only (Morii and Koga, 2003).

Archaetidylinositol phosphate (AI) synthase catalyzes the reaction where precursors L-*myo*-inositol-1-phosphate and CDP-archaeol are converted to AI phosphate as an intermediate which is further dephosphorylated to AI (Morii et al., 2009). This reaction is similar to the bacterial phosphatidylinositol phosphate (PI) synthase. Similar to AS and PS synthase, the AI and PI synthase show a broad substrate specificity accepting both, archaeal and bacterial lipid derivatives as substrates (Morii et al., 2014). Enzymes homologous to PS decarboxylase and phosphatidylglycerol (PG) synthase have been identified in archaea as AS decarboxylase and archaetidylglycerol (AG) synthase but not yet characterized biochemically (Daiyasu et al., 2005).

### SATURATION OF DOUBLE BONDS

The mature phospholipids of archaea exist in their fully saturated form. The archaeal enzyme digeranylgeranylglycerophospholipid reductase catalyzes the hydrogenation or saturation of the geranylgeranyl chains of unsaturated archaetidic acid (DGGGP) in a stereospecific manner (Xu et al., 2010). It belongs to the geranylgeranyl reductase (GGR) family that includes GGR from plant and prokaryotes that are mainly involved in photosynthesis. Prenyl reductases other than GGRs are also found in all three domains of life and these enzymes catalyze the complete or partial reduction of isoprenoid compounds like respiratory quinones, tocopherol, dolichol, and other polyprenols (Ogawa et al., 2014).

**FIGURE 4:**
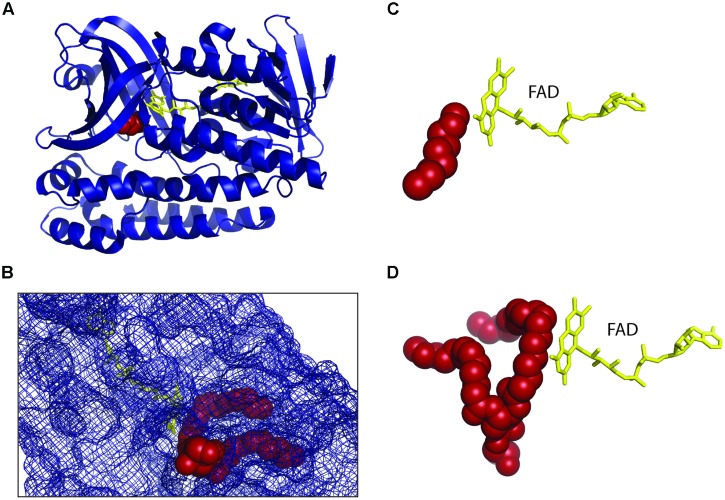
**Structure of *geranylgeranyl reductase* (GGR). (A)** The crystal structure of *Sulfolobus acidocaldarius* GGR at 1.85 Å resolution (PDB: 3ATQ) is shown (Sasaki et al., 2011) in complex with FAD (yellow). It is similar to the structure of GGR from *Thermoplasma acidophilum* (PDB: 3OZ2; Xu et al., 2010) sharing the FAD binding and the catalytic domain. Both the enzymes were crystallized where a lipid molecule (red) originating from the host organism was found in the substrate binding site. **(B)** The lipid binding cavity of GGR is R shaped having two tunnels where the larger tunnel B is more permissive and the smaller tunnel A is restricted in shape. One edge is located at the branching point of the tunnel. **(C,D)** Comparison of the ‘near perfect’ placement of the hydrocarbons of lipid moieties on the *re* face of the FAD from *S. acidocaldarius* GGR **(C)** and *Thermoplasma acidophilum* GGR **(D)**.

The structures of the archaeal GGR monomer from *Thermoplasma acidophilum* (Xu et al., 2010) and *Sulfolobus acidocaldarius* (Sasaki et al., 2011) show that they belong to p-hydroxybenzoate hydroxylase (PHBH) superfamily of flavoproteins (**Figure 4A**). The GGR from the thermophilic archaea *Thermoplasma acidophilum* was crystalized in complex with flavin adenine dinucleotide (FAD) where FAD adopts the close confirmation that possibly changes with the binding of the substrate, like in other members of the PHBH family. The reduction of FAD is brought about by either NADH or other reducing agents. Since the protein was overexpressed in *E. coli*, a surrogate lipid-like ligand assigned as phosphatidylglycerol (PGX) was found in the active site forming an imperfect fit to the substrate binding pocket. The lipid binding cavity of GGR is R shaped having two tunnels where the larger tunnel B is more permissive than the smaller tunnel A which is restricted in shape (**Figure 4B**). The *S. acidocaldarius* GGR is structurally similar to GGR from *Thermoplasma acidophilum* in FAD binding and the catalytic region (**Figures 4C,D**) but not in the C-terminal domain which is longer in *S. acidocaldarius* GGR. The conserved sequence motif (YxWxFPx7-8GxG) lies in the large cavity of the catalytic domain and is thought to keep the substrate in position for the reduction reaction as also indicated by mutational studies. Although the enzymes reduce GGGP, they also reduce the double bonds of related compounds like GGGP and GGPP (Sasaki et al., 2011). Another study where the *Methanosarcina acetivorans* GGR was expressed in *E. coli* along with four previous genes of the archaeal lipid biosynthetic pathway, the DGGGP derivative with a fully saturated isoprenoid chain could be obtained (Isobe et al., 2014). Interestingly, the saturation only took place when GGR was coexpressed with a ferredoxin gene found upstream of GGR in the genome of *Methanosarcina acetivorans*, the ferredoxin possibly functioning as a specific electron donor. However, no ferredoxin coexpression was required when the *Methanosarcina acetivorans* GGR was replaced by *S. acidocaldarius* GGR in the same study. Also, the conservation of the ferredoxin gene upstream of GGR in other archaea was not analyzed.

It is not known at what step of the biosynthetic pathway, hydrogenation takes place. However, since that CarS is specific for the unsaturated substrate, saturation probably takes place after the formation of CDP-archaeol. Although the enzyme AS synthase can accept both saturated and unsaturated substrates for catalysis, the detection of unsaturated AS in the cells of *Methanothermobacter thermautotrophicus* suggests that hydrogenation may already take place after the polar head groups are attached (Koga and Morii, 2007).

### TETRAETHER FORMATION

Tetraether (caldarchaeol) lipid structure with varying number (0–8) of cyclopentane moieties are widespread among archaea and a dominating membrane lipid structure in Crenarchaeota and Thaumarchaeota. Euryarchaeota synthesize archaeol or both archaeol and caldarchaeols. On the other hand, Thaumarchaeota have characteristic tetraether lipids with four cyclopentane moieties and a cyclohexane moiety (Villanueva et al., 2014). One of the most intriguing steps in the archaeal biosynthetic pathway is the tetraether formation. *In vivo* studies suggested that tetraethers are formed from saturated diethers via head to head condensation reaction. Pulse chase and labeling experiment of *Thermoplasma acidophilum* cells with [^14^C]-MVA showed that the label first incorporates into the archaeol until saturation and only then into caldarchaeol. When an inhibitor of tetraether lipid synthesis (terbinafine) is used, pulse labeling leads to the accumulation of diethers and this phenomenon can be reversed by removal of the inhibitor (Nemoto et al., 2003). However, in another study, radiolabelled archaeol was not incorporated into the tetraethers of *Methanospirillum hungatei* cells and the presence of double bonds was necessary for the incorporation of labeled DGGGP into the tetraethers of *Methanobacterium thermoautotrophicus* cells (Poulter et al., 1988; Eguchi et al., 2000). The enzyme responsible for the formation of the presumed and unusual C–C bond for tetraethers has not been identified and there is no* in vitro* data to support this hypothesis (Koga and Morii, 2007). Recently, an alternative pathway for tetraether and cyclopentane ring formation was hypothesized (Villanueva et al., 2014). A multiple lock and key mechanism was proposed owing to the ‘greater functional plasticity’ of the enzymes IPP synthase, GGGP synthase, and DGGGP synthase so that they can accommodate prenyl substrates with a ring structure and chain length longer than C20. The cyclopentane rings would be formed early in the pathway before attachment of the glycerol moiety and the C20 geranyl molecules would couple together via a tail-to-tail mechanism to form the C40 phytoene chain by phytoene synthase, an enzyme that is wide spread in archaea. However, both possibilities still need to be experimentally demonstrated.

## PHYSICOCHEMICAL PROPERTIES OF ARCHAEAL LIPIDS

### ARCHAEAL MEMBRANE LIPID COMPOSITION – RESPONSE TO STRESS

**FIGURE 5:**
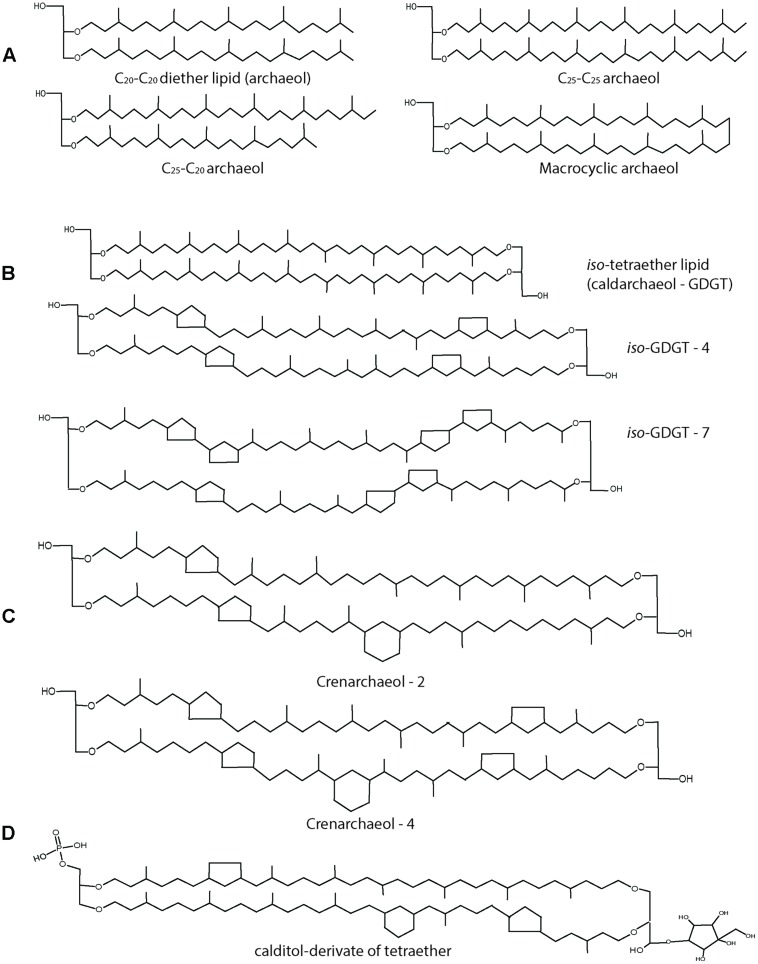
**Structures of archaeal lipids. (A)** Diether lipids (archaeol) with modifications in chain length and with macrocyclic ring structure are mostly found in Euryarchaeota. **(B)** Tetraether lipids (caldarchaeol) can contain up to 8 cyclopentane ring moieties. **(C)** Crenarchaeol lipid structure with cyclopentane and cyclohexane rings found only in Thaumarchaeota. **(D)** Calditol derivative of tetraether lipid found in *Sulfolobus spp.*

Within the archaeal lipids there is a great diversity varying in length, composition and configuration of the side chains (**Figure 5**). The most common archaeal core lipid is *sn*-2,3-diphytanylglycerol diether, generally called archaeol, which can undergo several modifications including hydroxylation and condensation. Pioneering studies on archaeal membrane lipid composition and biosynthesis have been performed in early eighties and nineties especially in halophiles using* in vivo* labeling experiments by Kates and colleagues (Kates, 1992, 1993; Kamekura and Kates, 1999) and reviewed in detail (Koga and Morii, 2007). Among others, the studies showed that halophiles are mainly characterized by the phospholipid known as phosphatidylglycerol methyl phosphate (PGP-Me) along with sulfated and desulfated archaeols (Kates, 1993). Archaeol bearing elongated hydrocarbon chains (C20–C25) are found in some methanogens and halobacteriales (**Figure 5A**; Koga et al., 1993; Kamekura and Kates, 1999). A seemingly head-to-head condensation of two diether lipid molecules is one of the most frequent and functionally important structural variations that leads to a glycerol-dialkyglycerol tetraether lipid, known as caldarcheol. It should be stressed, however, that the enzymatic mechanism resulting in this lipid species is entirely unresolved. This core lipid is the most widespread in Archaea, characterized by different modifications depending on the archaeal species. It is in particular abundant among the phyla Euryarcheaota, Creanarchaeota, and Thaumarchaetoa. Up to 8 cyclopentane moieties can be found in lipids of the Thermoplasmatales (**Figure 5B**) and in the Euryarchaeota phylum, in general (Macalady et al., 2004; UDA et al., 2004; Schouten et al., 2013). Interestingly, the presence of cyclopentane and cyclohexane ring is a distinct feature of Thaumarchaeota leading to a structure known as creanarcheol (**Figure 5C**; Pitcher et al., 2010; Damsté et al., 2012). In some thermoacidophiles and methanogens, a polar head group called nonitol is found which is composed of nine-carbon chain. Recent studies have revealed that the 9-carbon nonitol structure is often found as a polyhydroxylated cyclopentanic form called calditol (Koga and Morii, 2005). Therefore, these structure are now known as glycerol-dialkyl-calditol-tetraether (**Figure 5D**) and are the major component of the membrane of *Sulfolobales* species (Sugai et al., 1995; Untersteller et al., 1999; Gambacorta et al., 2002). The presence of tetraether lipids and the ratio of diether/tetraether lipids vary depending on the archaeal species and also upon growth conditions (Gambacorta et al., 1995). Likewise, there is also a wide diversity of polar lipids in archaea including phospholipids, glycolipids, phosphoglycolipids, sulpholipids, and aminolipids (Koga and Morii, 2005). The occurrence of different polar head groups depends on the archaeal family and can be used as unique taxonomic marker (Ulrih et al., 2009). Aminolipids, for instance, are prevalent in methanogens and are completely absent in halophiles and thermophiles (Gambacorta et al., 1995).

Bacteria and Eukarya use several mechanisms to maintain the membrane fluidity over a range of temperatures such the regulation of fatty acid composition adapting the degree of branching, saturation and chain length (Zhang and Rock, 2008). The homeoviscous adaption theory states that the lipid compositions in the membrane varies in response to environmental stresses in order to preserve a proper membrane fluidity (Oger and Cario, 2013). However, the exact changes in fatty acid composition in the membrane upon, for instance, a temperature shift differs from species to species. In *E. coli* the degree of fatty acid unsaturation increases along with a lowering of the temperature while some Bacillus species increase the amount of iso-fatty acids with the growth temperatures. The membrane also has to maintain its permeability barrier and in general it is believed that at the growth temperature, the lipid bilayer is a liquid crystalline state. The phase transition temperature at which the membrane is transferred from the crystalline into the liquid state is considered as a very important characteristic, and depends on the length of the hydrocarbon chains, the degree of saturation and the position of methyl groups; in Bacteria, the transition temperature ranges between -20 to up to 65∘C compared to archaea where the range is much wider between even up to 100∘C, a temperature where some archaea grow (Koga, 2012).

Archaea use different mechanisms to maintain the liquid crystalline phase over the entire growth temperature range. One control mechanism has been reported in the archaeon *Methanococcus jannaschii*, in which case the membrane properties are finely tuned by varying the ratio of diether, macrocyclic diether, and tetraether lipid (Sprott et al., 1991). In contrast, at higher temperatures, hyperthermophilic archaea may incorporate a higher degree of cyclopentane rings in their isoprenoid chain that increase the transition temperature. The presence of such lipid structures in archaea is an indication of a need to preserve the membrane function at the hostile environmental conditions. In particular, the presence of tetraether lipids and the chemically stable ether bonds are major adaptions (Jacquemet et al., 2009). The latter confers resistance to phospholipases attack from other organisms. Despite the general thought that the isoprenoid chains of the ether lipid are involved in thermal resistance, it has been shown that they are not absolutely required for tolerance to high temperature. Archaeol and caldarcheol were also found in the thermophilic methanoarchaeota (65∘C) and in mesophilic species (37∘C; Koga and Nakano, 2008). This suggests that the remarkable properties of the membranes of hyperthermophiles depend not exclusively on the tetraether composition of their lipids but that other aspects are involved as well. When fully stretched, the tetraether lipids span the entire membrane thickness leading to a monolayer, which is believed to stiffen the membrane in the presence of high growth temperatures (Kates, 1992; Jacquemet et al., 2009). This may also protect the membrane from possible lysis at high temperatures (Jacquemet et al., 2009). The presence of cyclic structures, in particular cyclopentene rings (De Rosa et al., 1980), is a hallmark for high growth temperatures and causes an increased membrane packing and thus a reduction in membrane fluidity (Benvegnu et al., 2008). However, also the characteristics of the polar head groups may influence membrane fluidity since the proper balance of negative and positive charges at the membrane surface is essential for its functioning. Therefore, varying the proportion of different polar head groups might be another way to response to external stresses (Oger and Cario, 2013). Furthermore, modification of the polar headgroups with carbohydrates increases hydrogen bonding between the lipids and thus will influence the stability of the membranes. In halophiles, the presence of glycerol methylphosphate attached to the archaeol moiety, contributes to the low membrane permeability under high salt concentration (Tenchov et al., 2006). A further example is cold adaption in the psychrophile archaeon *Methanococcoides burtonii*, where an increase in the degree of unsaturation in the isoprenoid chains allow growth at low temperatures as they exist in glacial environments (Russell and Nichols, 1999; Oger and Cario, 2013).

### *IN VITRO* BASED STUDIES ON THE STABILITY OF ARCHAEAL LIPIDS

Temperature impacts the membrane properties, influencing the ion permeability and phase behavior. It is believed that the unique membrane organization of archaeal tetraether lipid in a monolayer structure along with the presence of ether bonds renders such membranes, thermal resistance. Therefore, many studies have been conducted to understand the higher thermal stability of archaeal membrane lipids compared to the bacterial phospholipids. By comparing liposomes made of a polar lipid fraction from *S. acidocaldarius* and liposomes prepared from a bacterial lipid (POPC) or a synthetic lipid with a phytanyl chain (DPhPC), the importance of the methyl branched isoprenoid chain in membrane stability has been examined (Chang, 1994). When incubated at 100∘C for approximately half hour, archaeal liposomes showed a very low ion leakage compared to POPC and DPhPC liposomes that collapse after a few minutes incubation at that temperature. Likewise, only very slow release (8–10%) of the fluorophore carboxyfluorescin (CF) was observed with liposomes composed of *S. acidocaldarius* lipids compared to *E. coli* liposomes (50%) over a time interval of 62 days, while liposomes composed of a lipid extract from the thermophile bacterium *B. stearothermophilus* showed an intermediate stability (Elferink et al., 1994).

The extremely high heat tolerance of archaeal liposomes open opportunities for biotechnological applications with the ability to be stable even after several autoclaving cycles, which might be exploited for biomedical uses. Autoclaving is a common and effective method for decontamination and the possibility to autoclave the archaeosomes vesicle for the drug delivery gives new prospects for the liposomes formulation. Several studies have been performed regarding the ability of archaeosomes to enhance the immune response when used as novel vaccine and drug delivery system (Sprott et al., 1997; Patel and Sprott, 1999; Patel et al., 2000). The superior adjuvant activity of archaeosomes compared to the liposomes evokes sufficient immunostimulation and sustained immune response against cancer or specific infectious diseases. Archaeosomes confer higher stability to the vesicles enhancing the fusion with the immune cells (Krishnan and Sprott, 2008). These properties are dependent on the type of polar head groups and degrees of glycosylation on archaeal lipids (Dicaire et al., 2010; Whitfield et al., 2010). An enhanced cytotoxic cell response was in fact observed in archaeosomes enriched with archaeatidylserine and archaeatidylethanomine due to their fusogenic nature (Dicaire et al., 2010; Sprott et al., 2012). The effective archaeosomes stability against autoclaving was tested showing a remarkable strength against 2–3 cycles of autoclaving at pH 4.0–10.0 (Brown et al., 2009). Besides the high heat tolerance, archaeal lipids are known to be resistant to conditions of extremely low pH and their low proton permeability contributes to maintaining a constant intracellular pH. Monolayer liposomes reconstituted from the lipid fraction of *S. acidocaldarius* indeed exhibited very low proton permeability even at higher temperatures (Elferink et al., 1994; Komatsu and Chong, 1998). However, at acidic conditions the archaeosomes appeared less resistant to autoclaving possibly because of a higher protonation of the polar head groups, influencing hydrogen-bonds formation among the lipids. Liposomes containing long sugar chains linked to the phospholipids show much lower proton permeability than liposomes composed of lipids with only one sugar unit. The glycolipids amount in the polar lipid fraction of *Thermoplasma acidophilum* increases at lower pH and this seems a general mechanism for acidophiles against the chemically unstable conditions (Shimada et al., 2008). The low permeability of such liposomes can be exploited for drug-delivery with the added value of a high resistance against phospholipase A2, B and pancreatic lipase (Choquet et al., 1994). Due to their high pH tolerance they can easily pass the gastro-intestinal tract without damage. Overall, these studies confirm that the presence of tetraethers in archaeal membranes confers these membranes with a remarkable stability against high temperatures, low pH, and high salt. The degree of hydrocarbon chain saturation, the features of the polar head groups and the presence of cyclopentane ring (Dannenmuller et al., 2000) appear of secondary importance in providing stability.

## THE LIPID DIVIDE

### PROSPECT OF LUCA WITH MIXED MEMBRANE LIPID COMPOSITION

During the last decades many theories have been proposed about the origin of life and how the differentiation between the three domains of life occurred. All of these theories accept the existence of the last universal common ancestor (LUCA), also known as LUCAS or cenancestor from which organisms have diverged. Particular attention has been given on the membrane composition of LUCA. Isoprenoids are involved in a wide range of functions, and found both in archaea and bacteria. Whereas fatty acid metabolism is also widely distributed, fatty acid biosynthesis seems underdeveloped in archaea and in some organisms even absent. Therefore, one of the hypothesis is that early life forms were dependent on the presence of membrane lipids with isoprenoid hydrocarbon core. However, the most divergent feature that is at the base of the Lipid Divide is the glycerophosphate backbone. Archaea contains G1P as glycerophosphate moiety while bacteria depend on G3P. These two compounds are synthetized by two different enzymes that are not evolutionary correlated (Koga et al., 1998).

**FIGURE 6:**
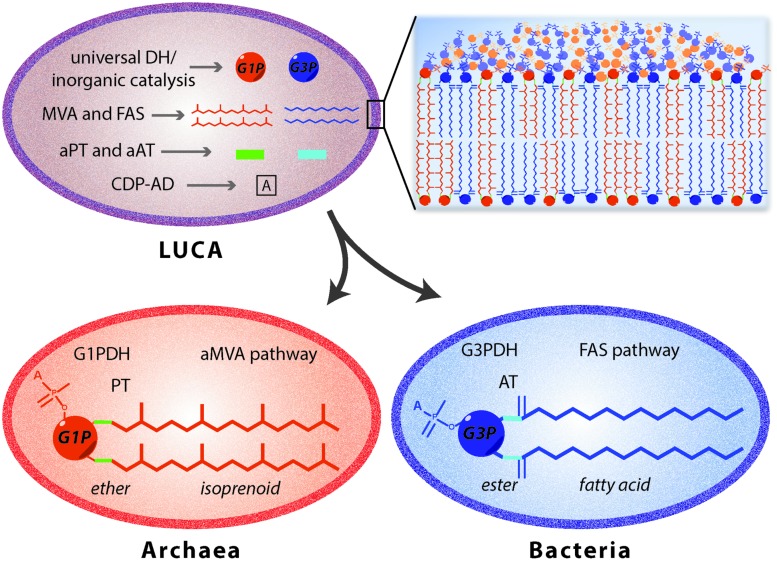
**Proposed evolutionary model for Archaea and Bacteria differentiation.** The existence of a cenancestor (LUCA) embordered by a mixed membrane is one of the main theories of organism’s evolution. This primordial organism would be characterized by the presence of heterogeneous enzymes involved in the synthesis of archaeal and bacterial lipids. Enzyme separation occurred by environmental pressures leading to the differentiation of bacteria with a phospholipid biosynthetic pathway (Yao and Rock, 2013) and archaea with ether-lipid biosynthesis. DH, dehydrogenase; MVA, mevalonate pathway; aMVA, alternate mevalonate pathway; FAS, fatty acid synthesis pathway; PT, prenyltransferases; AT, acyltransferases; ‘a’ used as prefix for PT and AT, ancestral; CDP-AD, CDP-alcohol phosphatidyltransferase family of enzymes.

Thus, different hypothesis were suggested to understand the process that brought the ancestor to archaeal, bacterial and eukaryotic organisms. According to Koga et al. (1998) the evolution of the two phospholipid pathways occurred independently leading to the simultaneous appearance of bacterial and archaeal enzymes. This contrast the theory of Martin and Russell (2003), according to whom the two different organisms evolved from an ancestor characterized by iron monosulphide compartment. Another hypothesis (Wächtershäuser, 2003) proposed a three stage process from the pre-cell to the eukaryotic cell. It was suggested that there was a cenancestor with a chemically derived heterochiral membrane containing both the enantiomeric forms of the glycerophosphate backbone, which slowly diverged into a more stable homochiral membrane organism leading to the differentiation between Bacteria and Archaea. This idea implies that such heterochiral mixed membranes are intrinsically unstable leading to the emergence of chiral selective enzymes and a divide between bacteria and archaea. However, hybrid membranes were tested for their stability using a mixture of egg phosphatidylcholine and extracted lipid from *Sulfolobus solfataricus* (Fan et al., 1995) and a higher stability compared to liposomes reconstituted of only archaeal lipid was observed. Moreover a heterochiral membrane consisting of bacterial G3P lipids and archaeal G1P lipids was analyzed (Shimada and Yamagishi, 2011) and the heterochiral membranes were found to be more stable at higher temperatures compared to liposomes prepared from only the bacterial lipids. Thus, based on these studies, the hypothesis of the existence of an ancestor with both the G1PDH and G3PDH would be possible but there must have been other factors that have driven the segregation into the two different domains. The theory of the coexistence of both the enzymes for the glycerophosphate enantiomers productions can be also extended to the other enzymes involved either in the synthesis of isoprenoid and fatty acid based phospholipids (**Figure 6**). In LUCA, four different membrane lipids may be obtained by the combination of the two glycerophosphate backbones with either isoprenoid or fatty acid hydrocarbon chains (Koga, 2011). Environmental pressure and the need to adapt to extreme conditions may have induced archaea to evolve their membranes (Valentine, 2007; Lombard et al., 2012b). In particular, the use of different hydrocarbon chains in response to the growth environment may have induced the segregation in Archaea and Bacteria and resulted in homochiral membrane formation (Koga, 2014).

### DIFFERENTIATION OF MEMBRANE LIPIDS IN ARCHAEA AND BACTERIA

The structural variability found in the membrane lipid composition of archaea and bacteria reflects differences and similarities in the respective biosynthetic pathways. When compared with the well-characterized bacterial ester-lipid biosynthetic pathway, several similarities with the archaeal ether-lipid biosynthesis are evident.

First, the sequence of reactions that yield the final membrane lipid from the building blocks is basically the same even though some enzymes involved in these reactions are equipped with specific features to the lipid of the two different domains. Second, the glycerophosphate backbone in both cases is synthetized by a reduction of DHAP at the 2-OH using NADH as cofactor while the two hydrocarbon chains are linked to the same position on the glycerol moiety. Third, the polar head attachment takes place via a CDP-activated intermediate (Koga, 2014). In particular the peculiar features that distinguish archaeal lipids from bacterial ones occur in the first half of the biosynthetic pathway while the last stages, which involve the replacement of the CDP GROUP with one of the polar groups, is essentially similar in these two domains of life. For the latter reactions, the enzymes belong to the same superfamily and share sequence similarity (Daiyasu et al., 2005; Koga, 2011). On the other hand, the isoprenoid building blocks synthesis differs in Bacteria and in Archaea since it takes place via two different pathway, the DOXP and the alternate MVA pathway, respectively (Koga and Morii, 2007). The other striking difference involved in the lipid divide is the enantiomeric configuration of the glycerophosphate backbone along with the saturation of the double bonds present on the isoprenoid chains and further modification of the hydrocarbon chains. The remarkable similarities of both biosynthetic pathways are an indication of the existence of a common ancestor with promiscuous enzymatic composition that sufficed for the synthesis of heterochiral membrane lipids.

## REVERSING EVOLUTION – SYNTHESIS OF ARCHAEAL LIPIDS IN BACTERIA?

Did a complex heterochiral membrane ever exist? There are several speculations about the membrane lipid composition of LUCA (Lombard et al., 2012b) and one evolutionary way to approach this is to design a cell which has a heterochiral membrane. The prospect of engineering *E. coli* with membranes harboring archaeal lipids has been initiated in several studies. In the first study, five genes were overexpressed in *E. coli*, four from a hyperthermophilic archaeon *Archaeoglobus fulgidus* (G1P dehydrogenase, GGPP synthase, GGGP synthase and DGGGP synthase) and one from *E. coli* (IPP isomerase; Lai et al., 2009). The enzyme IPP isomerase catalyzes the isomerization of IPP and DMAPP. Together with GGPP synthase, the carbon flux could be increased toward the synthesis of GGPP building blocks which had been demonstrated previously for carotenoid production in *E. coli* (Wang et al., 1999). The activity of each enzyme was monitored by different methods. IPP isomerase and GGPP synthase were analyzed by lycopene based calorimetric assay, G1P dehydrogenase activity was measured spectrophotometrically to detect DHAP dependent NADH oxidation reaction and using radiolabeled substrates, GGGP synthase activity was measured by thin layer chromatography (TLC) and high performance liquid chromatography (HPLC). To detect the formation of DGGGP in *E. coli*, lipids were extracted and dephosphorylated from a 24 h growing culture and analyzed by liquid chromatography mass spectrometry (LC-MS/MS) using electron spray ionisation (ESI-MS). This study showed that indeed the archaeal lipid biosynthesis pathway is functional in *E. coli*. Unlike G1P dehydrogenase and GGGP synthase, DGGGP synthase protein could not be detected and the amount of DGGGP formed was not quantitated.

In another study, the same four genes but now from the methanogen *Methanosarcina acetivorans* were chosen as it grows at 37∘C and are readily overexpressed in *E. coli* (Yokoi et al., 2012). Activity of all the enzymes was monitored in a TLC based assay using radiolabeled substrates. The lipids were extracted from the recombinant *E. coli* cells and unlike in previous study, lipids were not dephosphorylated but analyzed directly using LC/ESI-MS. Interestingly, the result showed that DGGGP synthesized by the archaeal enzymes was further metabolized by *E. coli* endogenous enzymes to form a PG-type derivative of DGGGP which was named DGGGP-Gro. Two speculations were made regarding the endogenous enzymes that might have brought about the reaction. If it emerged from the phospholipid biosynthetic enzymes of *E. coli*, it would require three enzymes to recognize and accept the archaeal substrate, namely CdsA, *sn*-G3P transferase and the phosphatase. However, addition of CTP to their* in vitro* TLC based assay did not increase the amount of product formation and no other polar head group attachment was observed. The other possibility is that the *sn*-1-phosphoglycerol group from the osmoregulated periplasmic glucans was transferred to digeranylgeranylglycerol (DGGGOH) directly by the phosphoglyceroltransferase system. The estimated amount of total archaeal membrane lipids extracted from *E. coli* cells in this study was only 60 μg/g wet cells and at these levels it is not possible to study the influence of archaeal lipids on the physical properties of the cytoplasmic membrane. In a follow up study, GGR and ferredoxin (see section saturation of double bonds) were introduced along with the other genes from *Methanosarcina acetivorans* and expressed in *E. coli*. The formation of DGGGP-Gro was reduced yielding mostly saturated archaetidic acid in *E. coli* (Isobe et al., 2014).

The archaeal lipids have also been synthesized* in vitro* using a set of five purified enzymes, two from bacteria and three from archaea. A mutant of FPP synthase of *E. coli* was used that was shown previously to synthesize GGPP, G1P dehydrogenase was from *B. subtilis*, GGGP synthase from methanogen *Methanococcus maripaludis*, DGGGP synthase and CarS from the hyperthermophilic *Archaeoglobus fulgidus*. All enzymes were purified, and by using substrates IPP, FPP, DHAP, and NADH, the enzymes were shown to be able to synthesize CDP-archaeol in the presence of CTP, Mg^2+^ and detergent at 37∘C (Jain et al., 2014). The feasibility of synthesizing archaeal lipids in *E. coli* and* in vitro* are promising first steps toward deciphering the biosynthetic pathway further and eventually understanding the properties of a cell with a heterochiral membrane lipid composition.

## FUTURE CHALLENGES

Although during the last decade, many of the intimate features of the archaeal lipid biosynthesis pathway have been resolved, there are still several important questions that need to be answered. Understanding the mechanism of tetraether formation and identifying the enzyme(s) involved in the reaction requires thorough investigations. *In vitro* analysis of such a reaction(s) would be great advancement in the field. Various other derivatives of diether and tetraether lipids like cyclopentane and macrocyclic ring formation, glycosylation and formation of crenarchaeol are also not well understood. The pathway refractory of archaeal lipid biosynthesis in *E. coli* is currently incomplete where the amount of archaeal lipids formed in comparison to the *E. coli* lipids is very low. Challenging aspect is to modulate and suppress the endogenous pathway and integrate the archaeal lipid biosynthesis pathway in the genome of the host yielding the exclusive formation of these lipid species. Further structural and biochemical analysis of the enzymes of the pathway from different families of archaea would progress the field and bring it in par with the understanding of bacterial phospholipid biosynthesis. Also, regulation of the phospholipid metabolism is poorly understood and could be enhanced through the use of genetic studies, which now became feasible because of the rapid developments in genetic toolbox in archaea. However, to study essential genes using these techniques is still a challenge.

Recent studies have shown that in spite of the uniqueness of the archaeal membrane lipid structure, they are not as distinct as previously thought. The presence of fatty acids and isoprenoids in the three domains of life and the common mode of polar head group attachment in bacteria and archaea, and the presence of homologues of archaeal G1P dehydrogenase and GGGP synthase in bacteria are a few of the similarities. Other question that still remains unanswered is the exact reason why G1P is ether linked to the isoprenoid hydrocarbon chains and G3P is linked via ester bonds to the fatty acid chains; are there still organisms with such mixed membranes? In order to answer such questions, more biochemical and functional investigation are needed on the archaeal lipid biosynthetic pathway along with a deep phylogenetic analysis.

## Conflict of Interest Statement

The authors declare that the research was conducted in the absence of any commercial or financial relationships that could be construed as a potential conflict of interest.
